# Soluble peptidoglycan fragments produced by *Limosilactobacillus fermentum* with antiproliferative activity are suitable for potential therapeutic development: A preliminary report

**DOI:** 10.3389/fmolb.2023.1082526

**Published:** 2023-02-15

**Authors:** Virginia Fuochi, Mariarita Spampinato, Alfio Distefano, Angelo Palmigiano, Domenico Garozzo, Chiara Zagni, Antonio Rescifina, Giovanni Li Volti, Pio Maria Furneri

**Affiliations:** ^1^ Dipartimento di Scienze Biomediche e Biotecnologiche (BIOMETEC), Università di Catania, Catania, Italy; ^2^ Center of Excellence for the Acceleration of Harm Reduction (CoEHAR), University of Catania, Catania, Italy; ^3^ CNR, Institute for Polymers, Composites and Biomaterials (IPCB), Catania, Italy; ^4^ Dipartimento di Scienze del Farmaco e della Salute, Università di Catania, Catania, Italy

**Keywords:** *Limosilactobacillus fermentum*, soluble peptidoglycan fragments, antiproliferative activity, colorectal cancer, amino acids, antibacterial activity

## Abstract

Currently, the use of probiotic strains and their products represents a promising innovative approach as an antagonist treatment against many human diseases. Previous studies showed that a strain of *Limosilactobacillus fermentum* (LAC92), previously defined as *Lactobacillus fermentum*, exhibited a suitable amensalistic property. The present study aimed to purify the active components from LAC92 to evaluate the biological properties of soluble peptidoglycan fragments (SPFs). The cell-free supernatant (CFS) and bacterial cells were separated after 48 h of growth in MRS medium broth and treated for isolation of SPFs. Antimicrobial activity and proliferation analysis on the human cell line HTC116 were performed using technologies such as xCELLigence, count and viability, and clonogenic analysis. MALDI-MS investigation and docking analysis were performed to determine the molecular structure and hypothetical mode of action, respectively. Our results showed that the antimicrobial activity was mainly due to SPFs. Moreover, the results obtained when investigating the SPF effect on the cell line HCT116 showed substantial preliminary evidence, suggesting their significant cytostatic and quite antiproliferative properties. Although MALDI was unable to identify the molecular structure, it was subsequently revealed by analysis of the bacterial genome. The amino acid structure is called peptide 92. Furthermore, we confirmed by molecular docking studies the interaction of peptide 92 with MDM2 protein, the negative regulator of p53. This study showed that SPFs from the LAC92 strain exerted anticancer effects on the human colon cancer HCT116 cell line *via* antiproliferation and inducing apoptosis. These findings indicated that this probiotic strain might be a potential candidate for applications in functional products in the future. Further examination is needed to understand the specific advantages of this probiotic strain and improve its functional features to confirm these data. Moreover, deeper research on peptide 92 could increase our knowledge and help us understand if it will be possible to apply to specific diseases such as CRC.

## 1 Introduction

Currently, natural substances are considered a promising alternative to conventional therapies (Yuan et al., 2016; Kim et al., 2018; Kim et al., 2021; Lim et al., 2021; Kim et al., 2022). Among the macromolecules that play an essential role in cell growth and development, closely related to physiological functions are the bacterial cell wall peptidoglycan (PG) components (Wheeler et al., 2014; Semenov, 2021). The PG polymer is composed of *N*-acetylmuramic and *N*-acetylglucosamine sugar chains alternated with cross-linking peptide chains containing d- and l-amino acids. The polysaccharides and teichoic acids in the cell wall are covalently linked to PGs (Chapot-Chartier and Kulakauskas, 2014). As a whole, it is a dynamic and complex structure, with various chemical modifications and trimming mechanisms that produce disaccharide-containing elements (Bersch et al., 2021). In addition to playing an essential role in bacterial physiology and maintaining the cell’s shape and integrity, it acts as an interface between the bacterium and its environment. In fact, recently, attention toward it has been increasing due to its various pharmacological activities, such as antitumor effects (He et al., 2017; Wang et al., 2018).

Colorectal cancer (CRC) is the third most common cancer and the fourth most common cause of cancer-related death (McGuire, 2016). While environmental and genetic factors play a significant role in the pathogenesis of colon cancer, extensive research has suggested that nutrition and dysbiosis may play both a causal and protective role in the development of this cancer (Koliarakis et al., 2019; Thanikachalam and Khan, 2019). Indeed, Western dietary habits, obesity, and heavy alcohol consumption play significant roles in causing CRC (Cheng et al., 2020). Moreover, several bacterial species have been associated with CRC, such as *Streptococcus bovis* (Gupta et al., 2010), *Bacteroides fragilis* (Chung et al., 2018), *Fusobacterium nucleatum* (Bashir et al., 2015), and *Peptostreptococcus anaerobius* (Long et al., 2019; Cheng et al., 2020). On the contrary, several studies have demonstrated that probiotic bacteria can be effective for various medical conditions, including CRC (Ishikawa et al., 2005; Uccello et al., 2012; Chong, 2014; Fong et al., 2020).

In this context, many studies have shown that some *Lactobacillus* species, such as *Lactobacillus casei*, have remarkable antitumor activity both *in vivo* and *in vitro* (Kato et al., 1981; Yasutake et al., 1984; Shimizu et al., 1987; Kato et al., 1988; Matsuzaki et al., 1996). The antiproliferative activity appears due to mechanisms involving activated macrophages, modulation of the host’s immune response, and regulation of cellular apoptosis (Lidbeck et al., 1992; Saha and Saroj, 2022). Several lactobacilli are claimed to be health-benefiting maintaining intestinal microbial balance, protecting against the invasion of pathogenic microorganisms, and activating the innate immunity response of the host (Veiga et al., 2020). Therefore, some of these strains are currently used as probiotic supplements in technological additives and for therapeutic purposes (E.F.S.A., 2010; E.F.S.A., 2011a; E.F.S.A., 2011b; E.F.S.A., 2011c; E.F.S.A. and Panel, 2013; E.F.S.A. et al., 2021).

Extracts and fragments of peptidoglycan can induce apoptosis in several cell cancer lines (Troll et al., 2009; Vazquez-Sanchez et al., 2014; Wang et al., 2018; Patra et al., 2022). The principal tumor suppressor protein is p53, which induces cell death by apoptosis in response to various stress conditions (Aubrey et al., 2018). p53 binds explicitly to a 20-base pair (bp) consensus DNA sequence, acting as a transcription factor activator and leading to cell growth suppression and cell death (Kitayner et al., 2010). Inactivation of p53 is the most prevalent defect in human cancers.

p53 is negatively regulated by the oncoprotein MDM2 (murine double minute 2) (Zhao et al., 2014). MDM2 is an E3 ubiquitin ligase that targets the degradation of the p53 tumor suppressor and inhibits the expression of p53-related genes.

Since it has been discovered that MDM2 and p53 are part of an auto-regulatory feedback loop, a new approach for cancer treatment consists of stimulating p53 by inhibiting its interaction with MDM2 (Wu et al., 1993). It was already reported that compounds that activated the p53 pathway by inhibiting the *mdm2* mRNA level execute anti-HCT116 cancer activity (Zhang et al., 2022).

Our previous studies showed that strains of *Lactobacillus,* isolated from clinical samples, exhibited suitable amensalistic properties, and their purified components have biological properties (Fuochi et al., 2018; Fuochi et al., 2019; Stivala et al., 2021). The present paper describes the production and isolation of the active fragments of peptidoglycan, belonging to a strain of *Limosilactobacillus fermentum,* responsible for the antimicrobial activity and antiproliferative events observed in tumor cells. Subsequently, an analysis of the bacterial genome was performed by searching for the *mur* genes to confirm the structure of the peptide fragment since MALDI investigations had failed to detect it. The amino acid structure is called peptide 92. Moreover, we performed docking analysis to confirm the hypothesis that the antiproliferative effects exerted by peptidoglycan fragments were due to the interaction of peptide 92 with MDM2, preventing the binding of the protein with p53. In fact, inhibitors of the Mdm2–p53 interaction, which restore the functional p53, constitute potential non-genotoxic anticancer agents with a novel mode of action. Taken together, our results provided a scientific reference for further investigations and applications in discovering natural antitumor drugs from lactic acid bacteria.

## 2 Materials and methods

### 2.1 Bacterial strain

For this purpose, the strain of *Limosilactobacillus fermentum*, LAC92 (GenBank ID:CP021790.1), earlier defined as *Lactobacillus fermentum* and isolated from the oral cavity, as previously described (collection of the Laboratory of Applied Microbiology, Department of Biomedical and Biotechnological Sciences, Università degli Studi di Catania, Italy), was used (Fuochi et al., 2017).

LAC92 was grown in de Man, Rogosa, and Sharpe (MRS) broth (Oxoid, Thermo Fisher Scientific Inc., Rodano (MI), Italy, CM0359) at 37°C for 48 h under microaerobic conditions before proceeding with subsequent investigations.

### 2.2 Composition of the culture media

The MRS broth used had the following components per liter: peptone, 10.0 g; yeast extract, 4.0 g; `Lab-Lemco’ powder, 8.0 g; glucose, 20.0 g; sodium acetate 3 H_2_O, 5.0 g; K_2_HPO_4_, 2.0 g; triammonium citrate, 2.0 g; MgSO_4_ 7 H_2_O, 0.2 g; MnSO_4_ 4 H_2_O, 0.05 g; sorbitan mono-oleate, 1 mL; pH 6.2 ± 0.2 at 25°C. The medium was sterilized by heating to 121°C for 20 min.

### 2.3 Isolation and storage of SPFs

For isolating soluble peptidoglycan fragments (SPFs), a modified method of Fichera et al., (2016) was performed. Briefly, bacterial cells were collected by centrifugation at 7000 g for 15 min at room temperature, resuspended in 500 mL sterile PBS containing 2.5 g of d-glucose and 5 mg of penicillin G, and incubated under agitation at 32°C for 30 min. The supernatant containing soluble peptidoglycan fragments was then harvested by centrifugation at 12,000 *g* for 30 min at 4°C, then heated at 65°C for 15 min, and finally concentrated at 4°C by flash evaporation.

CFS and the bacterial pellet discarded from the isolation process of SPFs were used for further investigation.

All products were immediately used for the assays described. Furthermore, SPFs were kept at +4°C and −30°C to carry out stability tests at 30 days and again at 90 and 180 days.

### 2.4 *In vitro* antimicrobial activity

The CFS, bacterial pellets, and SPFs obtained were tested against several pathogens. CFS was neutralized with NaOH 1 mol/L at pH 7.0 before use. The inhibitory activity was performed by the agar-well diffusion assay and microdilution broth method as described by CLSI (C.L.S.I., 2018). Antibacterial activity was evaluated against *Escherichia coli* ATCC 25922, *Enterococcus faecalis* ATCC 29212, *Sarcina lutea* ATCC 9341, *Staphylococcus aureus* ATCC 25923, *Pseudomonas aeruginosa* ATCC 27853, *Klebsiella pneumoniae* ATCC 70060, and *Acinetobacter baumannii* ATCC 19606.

Briefly, the substances (CFS, pellet, and SPF) were added to wells in pre-inoculated MH agar plates for an agar-well diffusion assay (Stivala et al., 2021). MH plates were incubated at 37°C overnight in aerobic conditions. Finally, the inhibition zones were measured in millimeter by a gauge. Results were expressed in mm ± SD.

Regarding the microdilution broth method, minimum inhibitory concentration (MIC) values were determined according to CLSI. The assay was performed in 96-well polystyrene plates (Corning® 96-well microplates) with CAMHB medium (Cationic Adjusted Muller Hinton Broth; Oxoid). Briefly, 100 µL of the medium was added to each well, and 100 µL of the test substance was added along the first column. The dilutions (50%–0.1%) were carried out by direct microdilution in the plate. No substance was added in the last two columns. Then, a microbial suspension was made for each strain under examination, and the broth dilutions were prepared to obtain the final concentration of 10^4^–10^5^ CFU/mL. The last column was filled with fresh medium (negative control), while in the penultimate column, the tested pathogen was inoculated (positive control). Finally, microplates were incubated at 37°C overnight, and the MIC value was defined as the lowest concentration that inhibited the pathogenic strains’ visible growth. Moreover, the minimum bactericidal concentration (MBC) was performed after the MIC assay had been completed. Briefly, the dilution representing the MIC and two more concentrated dilutions were plated on MHA and enumerated to determine viable colonies after incubation at 37°C overnight. The MBC is the lowest concentration that demonstrates a pre-determined reduction (99.9%) in CFU/mL compared to the MIC. Results were expressed as % v/v (Fuochi et al., 2021b).

### 2.5 *In vitro* antiproliferative evaluation of the HCT116 cancer cell line

#### 2.5.1 Cell culture

Human colorectal cells HCT116 (ATCC® CCL-247™) were purchased from ATCC Company (Manassas, Virginia, United States). Cells were suspended in RPMI 1640 (Gibco, Cat. No. 21870076) culture medium containing 10% fetal bovine serum (FBS, Gibco, Cat. No. 10082147), 100 U/mL penicillin, and 100 U/mL streptomycin (Gibco, Cat. No. 15070063). At 80% confluency, cells were passaged using a trypsin–EDTA solution (0.05% trypsin and 0.02% EDTA, Gibco, Cat. No. 25300054).

#### 2.5.2 Real-time monitoring of cell proliferation

xCELLigence experiments were performed using the RTCA (Real-Time Cell Analyzer) DP (Dual Plate) instrument according to the manufacturers’ instructions (Roche Applied Science, Mannheim, Germany and ACEA Biosciences, San Diego, CA). The RTCA DP instrument includes three main components.

RTCA DP Analyzer, which is placed inside a humidified incubator at 37°C with 5% CO_2_,
RTCA Control Unit with RTCA software, and
EPlate 16.

The optimal seeding number was determined by cell titration and growth experiments. After seeding the optimal cell number (3.0 × 10^3^ cells per well), cells were automatically monitored every 20 min for 65 h. The optimal cell number was determined in a preliminary set of experiments to obtain a significant CI (Cell Index) value and constant cell growth during the entire duration of the experiment (Castruccio Castracani et al., 2020).

#### 2.5.3 Count and cell viability

Cell viability measurements were performed by a Muse® Cell Analyzer after 24 and 48 h (Merck Millipore Corporation, Milan, Italy) as previously described by Fuochi et al. (2017). The measurement was based on the differential permeability of two DNA-binding dyes. The nuclear dye stains only nucleated cells, while the viability dye brightly stains dying and dead cells. A volume of 180 μL of Muse® Count & Viability Reagent was added to 20 μL of suspended cells. Cells were incubated at room temperature for 5 min, and then the measurements were performed.

#### 2.5.4 Cell migration

Cell proliferation and migration were studied using the “wound healing” assay. Cells were seeded separately in 6-well dishes and cultured until confluence. Cells were scraped with a 200-μL micropipette tip and monitored at five time-points (0, 6, 24, 30, and 48 h). The uncovered wound area was measured and quantified at different intervals using ImageJ 1.37v.

#### 2.5.5 Clonogenic assay

Colony assays were performed by seeding cells in 6-well plates at low density (2500 cells/well) and allowing growth for 9 days. Then, cells were washed once with PBS 1X and fixed with a solution of methanol/acetic acid (3:1) for 5 min. Furthermore, a solution of crystal violet was added to stain the colonies for 15 min. Finally, colony images were acquired using a Leica microscope.

### 2.6 MALDI-MS analysis

MALDI mass spectrometry analysis was performed on SPFs, while crude MRS broth and MALDI matrix preparations without the analyte were used as blanks.

Spectra were recorded using a 4800 Proteomics Analyzer MALDI-TOF/TOF mass spectrometer (Applied Biosystems, Framingham, MA) equipped with an Nd:YAG laser at a wavelength of 355 nm with a less than 500-psec pulse and 200-Hz firing rate. All measurements were performed in both negative and positive polarity reflector modes using CHCA and DHB matrices. External calibration was performed on a standard peptide mix (AB-SCIEX), and mass accuracy was 100 ppm in the MS mode and 0.25 Da in the MS/MS mode. Approximately 2000 laser shots were accumulated for each spectrum in the MS experiments; 4000 to 16,000 shots were summed for the MS/MS data acquisitions. The tandem mass spectra reported in this study were acquired with and without a collision gas (He, air at low pressure).

Peak lists were generated for every spectrum of different fractions, and peptide–signal candidates were selected (for MS/MS analysis) comparing SPF spectra with MRS crude broth (with no *Lactobacillus* strains) and with MALDI matrix blanks (DHB and CHCA 10 g/L in TFA 0.1%/ACN 3:2) so that the only signals observed exclusively in CFS fractions were fragmented.

Samples were analyzed before and after purification with C18 Zip Tips (following the manufacturer’s protocols) at different concentrations (dilutions 1:10 ÷ 1:500, solvent TFA 0.1%/ACN 3:2).

### 2.7 Determination of structural features by *mur* gene research

All *mur* genes in the LAC92 strain genome were searched. Indeed, the strain was previously sequenced by Illumina MiSeq and assembled by MIRA v 4.0.2. Moreover, the sequence was deposited in GenBank (ID:CP021790.1.) with the assembly identification code GCF_002192435.1.

### 2.8 Docking calculations

Molecular docking experiments of SPF (defined as peptide 92) with the selected protein were performed with AutoDock Vina provided in YASARA (v. 22.5.22, YASARA Biosciences GmbH, Vienna, Austria). The 2D chemical structure of peptide 92 was built using MarvinSketch (https://chemaxon.com/products/marvin) to obtain a 3D molecule from a 2D structure and saved in the pdb file format. The software supported the valence checking, atom and bond query, stereochemistry, and user-defined templates.

The protein crystal structure of MDM2-p53 (PDB ID: 1YCR) was collected from the Protein Data Bank (PDB, http://www.rcsb.org/pdb) and optimized using YASARA software (Krieger et al., 2002). A docking employing a cell with an extension encompassing all atoms spanning 5 Å from the exterior of the p53 structure was performed (Rescifina et al., 2014; Floresta et al., 2022). Screening uses YASARA structure software with the macro dock_runscreening.mcr set in the VINA method, runs = 100, and AMBER14 force field. All the parameters were inserted at their default settings, as previously reported (Varrica et al., 2018; Floresta et al., 2019; Crocetti et al., 2022). Docking results were analyzed using BIOVIA Discovery Studio.

### 2.9 Statistical analysis

All the experiments were carried out four times, each in triplicate. In addition, statistical analysis was performed using GraphPad Prism 8 software. Specifically, the unpaired Welch’s *t*-test was selected for two data groups. Likewise, the null hypothesis was tested by multiple comparison analysis of variance (ANOVA) to compare multiple treatment groups. Differences between experimental groups were determined considering the statistical significance *p* < 0.05. Data were reported as mean ± SD.

## 3 Results

### 3.1 Antimicrobial activity

The antagonistic activity of LAC92 components was assessed against the following target bacteria: *E. coli* ATCC 25922, *E. faecalis* ATCC 29212, *S. lutea* ATCC 9341, *S. aureus* ATCC 25923, *P. aeruginosa* ATCC 27853, *K. pneumoniae* ATCC 70060, and *A. baumannii* ATCC 19606. CFS and SPFs showed good activity both against Gram-positive and Gram-negative pathogens (Table 1). In particular, *S. lutea* showed the greatest sensitivity, followed by *E. coli* and *A. baumannii*. The strains that proved to be less sensitive, although still inhibited, were *E. faecalis* and *S. aureus*. No activity was shown by the LAC92 bacterial pellet. These results were also confirmed by the microdilution method (Table 2).

**TABLE 1:** Zones of inhibition caused by LAC92 components against human pathogens. Results are expressed in millimeter (diameter) ± SD.

LAC92 component	*E. coli*	*E. faecalis*	*P. aeruginosa*	*K. pneumoniae*	*S. lutea*	*S. aureus*	*A. baumannii*
CFS	18.5 ± 0.2	13.2 ± 0.3	17.3 ± 0.4	18.3 ± 0.2	25.0 ± 0.4	15.5 ± 0.3	16.4 ± 0.2
Bacterial pellets	≤6	≤6	≤6	≤6	≤6	≤6	≤6
SPFs	15.5 ± 0.1	≤6	15.4 ± 0.4	15.4 ± 0.3	22.4 ± 0.4	≤6	13.2 ± 0.3

≤6 = no zone of inhibition.

**TABLE 2:** Antibacterial activity of LAC92 components by the microdilution method. MIC and MBC values are expressed as % v/v.

LAC92 component	*E. coli*		*E. faecalis*		*P. aeruginosa*		*K. pneumoniae*		*S. lutea*		*S. aureus*		*A. baumannii*	
	MIC	MBC	MIC	MBC	MIC	MBC	MIC	MBC	MIC	MBC	MIC	MBC	MIC	MBC
CFS	25	25	25	25	12.5	25	50	50	12.5	50	25	50	25	25
Bacterial pellets	R	—	R	—	R	—	R	—	R	—	R	—	R	—
SPFs	50	100	25	50	25	50	50	100	25	50	50	100	50	100

R = resistant.

### 3.2 Biological effects of SPFs on the HCT116 cancer cell line

#### 3.2.1 xCELLigence real-time cell analysis

**FIGURE 1:**
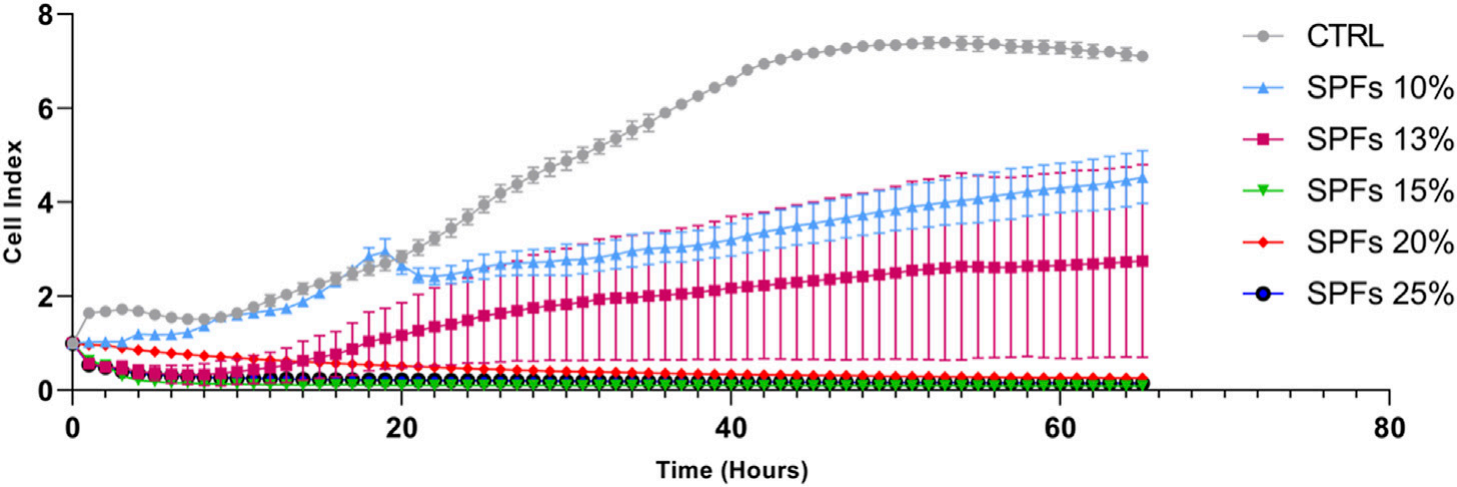
Real-time cell proliferation analysis at different concentrations (%v/v) of SPF (10%, 13%, 15%, 20%, and 25%) influencing cell index (standard cell index adhesion curve). From 15% onward SPF results are cytotoxic, while among the lower concentrations, the 13% deviates more from the cell index trend of the control. The curves represent the mean cell index value from 4 wells ±SD.

A preliminary investigation was performed by seeding cells at a defined concentration (3 × 10^3^ cells per well) and monitoring the growth using a live cell analysis system xCELLigence for 65 h. Specifically, this analysis allowed evaluating the dynamic cell index in real-time upon exposure to different concentrations (% v/v) of SPFs (10%, 13%, 15%, 20%, and 25%) in the growth medium. Data shown in Figure 1 demonstrate the cytotoxicity of SPFs at concentrations above 15%. Conversely, the first non-lethal but cytostatic concentration whose cell index was maintained at lower levels compared to those of the control was the 13% SPFs. For this reason, to use a non-lethal but compromising concentration, the treatment SPFs at 13% v/v were selected to perform all the following *in vitro* experiments.

#### 3.2.2 SPFs reduced cell count and viability

**FIGURE 2:**
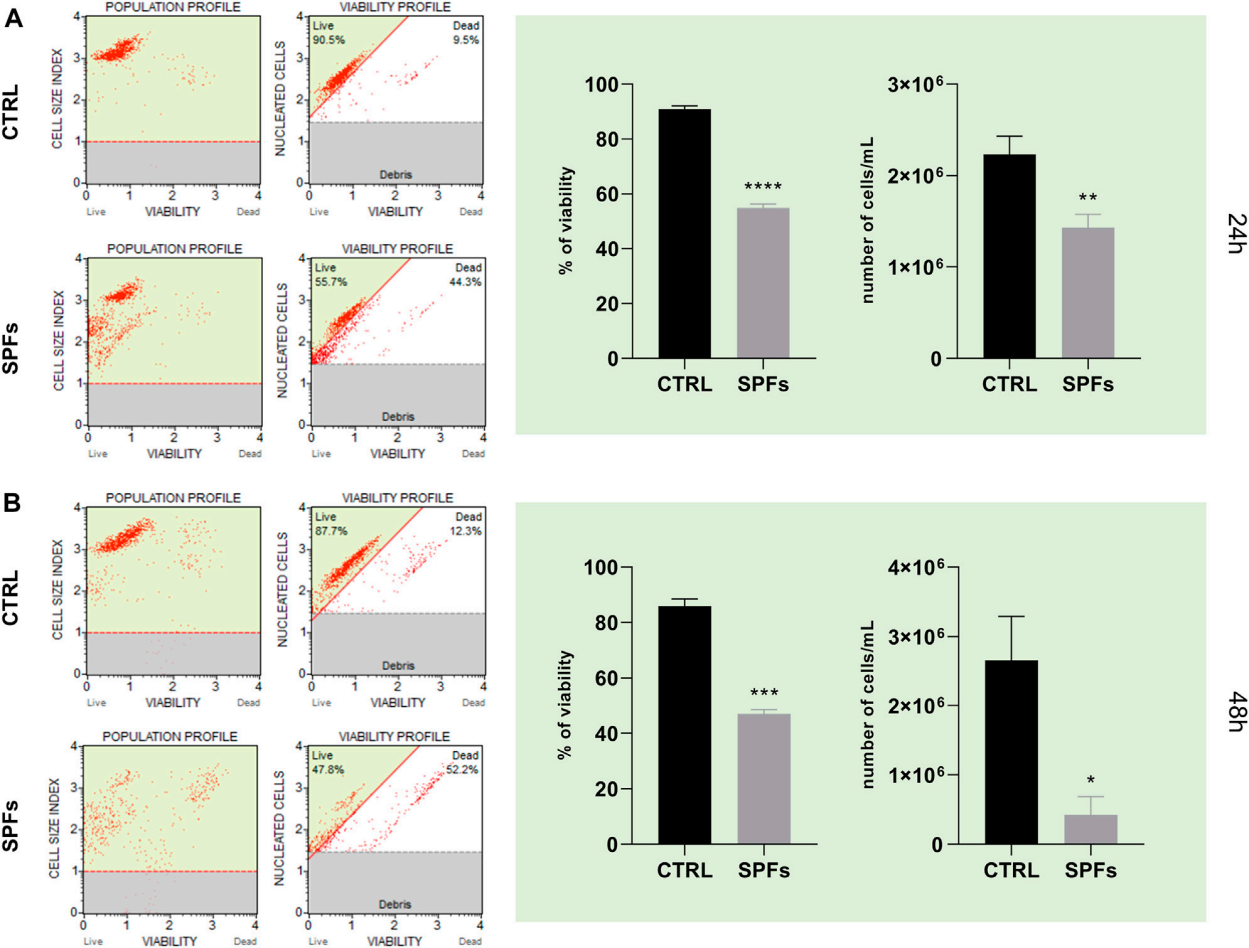
SPF affects cancer cell count and viability. **(A)** Cell population and viability profiles (from left to right) for CTRL and SPFs (from the top to bottom) after 24 h—histograms of percentage of viability and number of cells/mL of suspension after 24 h. **(B)** Cell population and viability profiles (from left to right) for CTRL and SPFs (from the top to bottom) after 48 h—histograms of percentage of viability and number of cells/mL of suspension after 48 h. Data are expressed as the means ± SD of three experiments performed in triplicate (*p < 0.05 *versus* CTRL).

As shown in Figure 2, cell count and viability assay were performed on the HCT116 cell line both at 24 and 48 h (panel A and panel B, respectively). In particular, the treatment with SPFs induced a significant decrease in both parameters after 24 h compared to the control. This evidence suggested a remarkable antiproliferative and cytotoxic effect of SPFs on the tumor cell model.

#### 3.2.3 SPFs affected cell migration and proliferation

**FIGURE 3:**
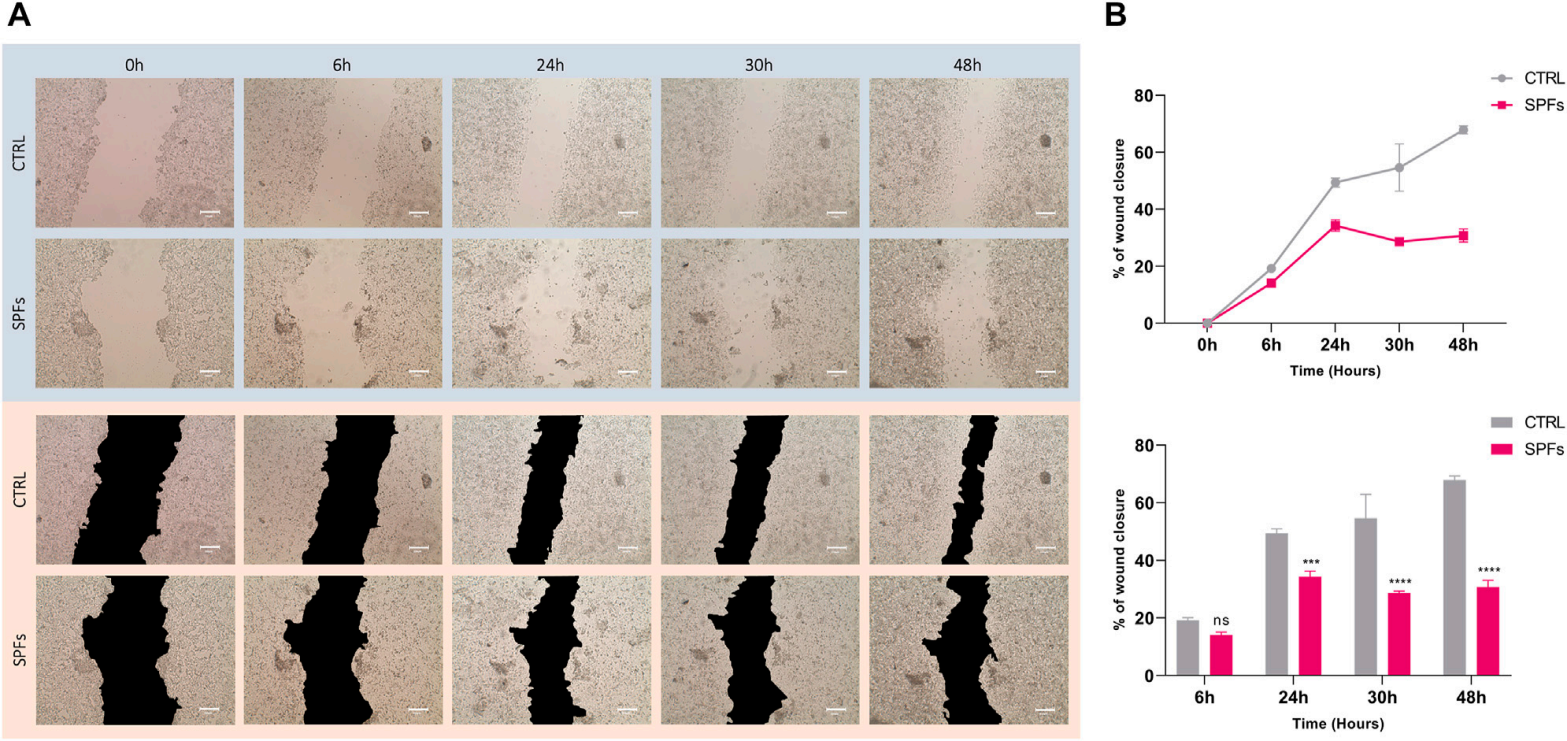
Inhibiting effects of SPFs on cancer cell migration. **(A)** Representative photos of wound healing assay with or without SPFs. Photos were taken from time 0–48 h (columns from left to right). All images were acquired using a Leica microscope with a magnification of ×20. The scale bar represents 200 µm. **(B)** Quantification of wound closure percentage expressed as a line graph (top) and bar chart (bottom). Data are expressed as the means ± SD of three experiments performed in triplicate (*p < 0.05 *versus* CTRL).

Based on previous analysis, cell proliferation was further evaluated by performing the wound healing assay. The wound closure area was quantified at different time-points from T_0_ (6, 24, 30, and 48 h) and compared between SPF treatment and the untreated control (see Figure 3A). The results show a decrease in the wound closure rate of SPFs compared to the control, related to the SPF’s ability to inhibit the proliferation and migration rate in HCT116. The same evidence is more clearly represented in Figure.3B.

#### 3.2.4 SPFs inhibited colony formation

**FIGURE 4:**
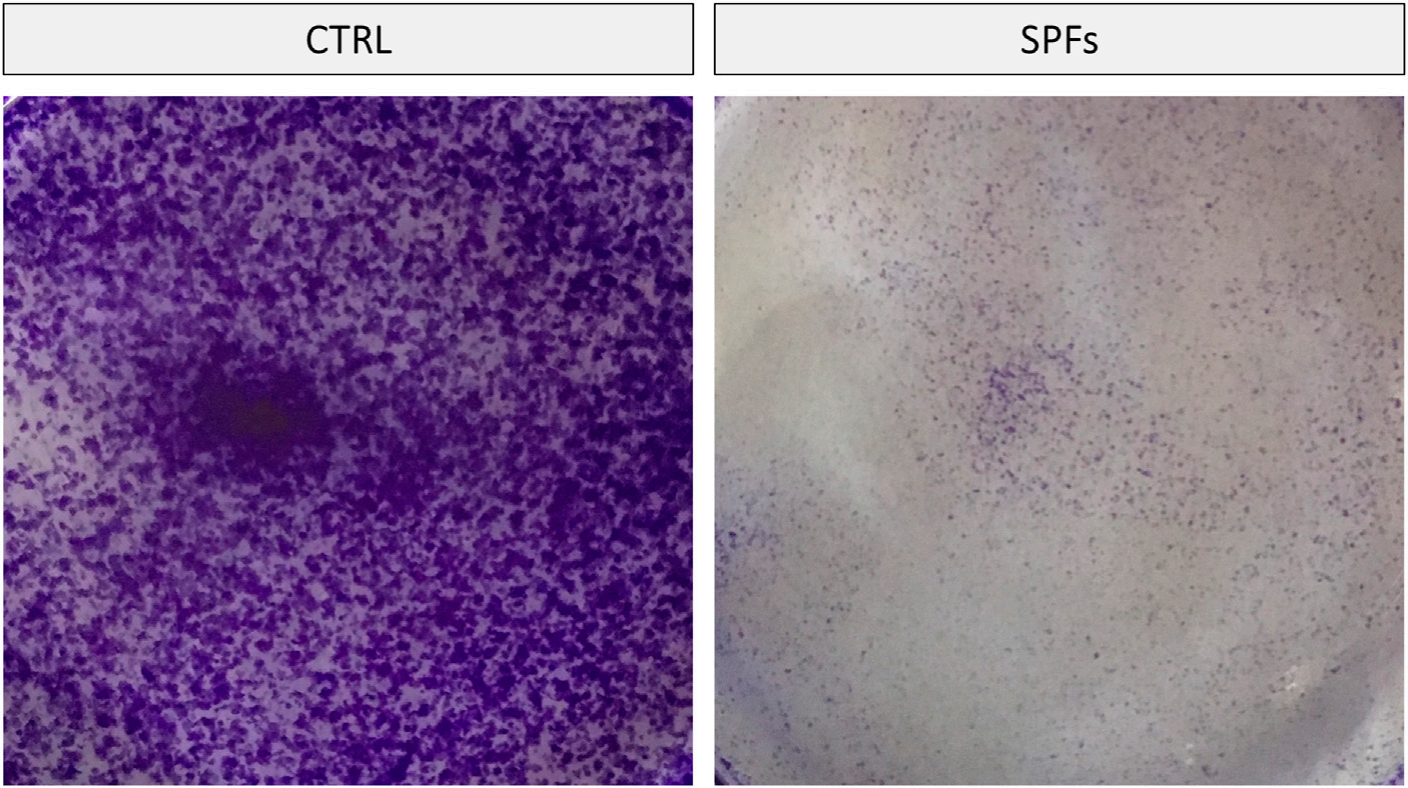
SPFs inhibited cancer cell capacity to form colonies. Representative photos of clonogenic assay performed using crystal violet staining. SPFs drastically reduced the number and size of cell colonies after 9 days of incubation compared to the untreated control.

In order to further evaluate the cytotoxic properties of SPFs in our cancer cell line, a clonogenic assay was performed. The data obtained showed a notable antiproliferative effect of SPFs compared to the control. Remarkably, the number of colonies was significantly reduced with SPFs, as reported in Figure 4.

### 3.3 MALDI-MS analysis

The most commonly employed MS-based strategy for identifying peptides employs low-energy collision-induced dissociation (CID) to produce characteristic fragments to determine peptide sequences. MALDI-MS/MS fragmentations generated by CID are typically dominated by cleavages along the peptide amide backbone, resulting in formation of a series of overlapping b- and y-type ions.

The first attempt to identify the possible peptide content of the SPF fraction consisted in searching protein databases (SWISS-PROT) of Gram-positive bacteria by setting the search criteria with no enzymatic cut, *Firmicutes* taxonomy, 150 ppm mass tolerance for precursors, and 0.25 Da mass tolerance for MS/MS fragments. However, this approach failed as databases containing information on the peptidoglycan composition of different bacterial strains have not yet been implemented. In addition, DB searches are based on homology, so unique species-specific sequences might not be identified, and this is particularly true for small and biologically active polypeptides (Medzihradszky and Chalkley, 2015).

For this reason, it was not possible to carry out a peptide mass fingerprinting search on the MALDI profiles of different soluble fractions of peptidoglycan nor was it possible to use methods such as an MS/MS ion search to search for peptide sequences by exploiting the fragmentation spectra of the signals present exclusively in the fraction CFS.

Bacterial PG hydrolases can cleave covalent bonds in peptidoglycan sacculi or their fragments (Vollmer et al., 2008). Bacterial PG hydrolases form a vast and highly diverse group of enzymes capable of cleaving bonds in polymeric PG and/or its soluble fragments. Each hydrolase class has cleavage site specificity, *e*.*g*., endopeptidases cleave peptide amide bonds, and *N*-acetyl-*β*-d-muramidases cleave the glycosidic bond between MurNAc and GlcNAc residues. Penicillin has the activity of PG hydrolases but not the specificity (of cleavage site).

The MS-based analysis of PG components is hampered by the diversity of penicillin treatment products; each can be originated from different cleavage sites of the cross-linked structure of PG. It is not possible to establish *a priori* the fragments produced, nor, through the knowledge of the amino acids involved in the peptidic part of the PG, to build a list of the expected products as is used in the classical proteomics approach.

However, CID can induce other fragmentation pathways that can potentially improve the confidence of peptide assignments, providing composition-specific information. One of these pathways results in the forming of internal immonium ions with the general structure RCH = NH_2_^+^ (where R is the amino acid side chain) and a mass of 27 Da less than the residue mass. These ions can be formed directly by fragmentation of the *N*-terminal residue of the peptide. Furthermore, they can also be formed by two cleavages surrounding a particular residue during CID, *e*.*g*., an a-type cleavage following either a b- or y-type cleavage.

In order to study the hypothetical amino acidic component of the SPFs, MS/MS spectra of putative peptides were analyzed by manual *de novo* sequencing; however, it was not possible to assign a unique sequence. The hypothesis of the existence in SPFs of a peptide/protein component, however, is corroborated by

1) The presence of (at least three) immonium ions (and related ions) in the low-mass region of spectra.
2) MS/MS spectra show a2 and b2 ion fragments as well as y2 fragments.
3) Loss of GlcNAc or MurNAc residues from the molecular ion.
4) Accurate delta masses of amino acid residues from the low-to high-mass region of the spectra.
5) The absence of these signals in MRS broth and DHB and CHCA blanks.
6) The presence of these signals in both DHB and CHCA preparations.

**FIGURE 5:**
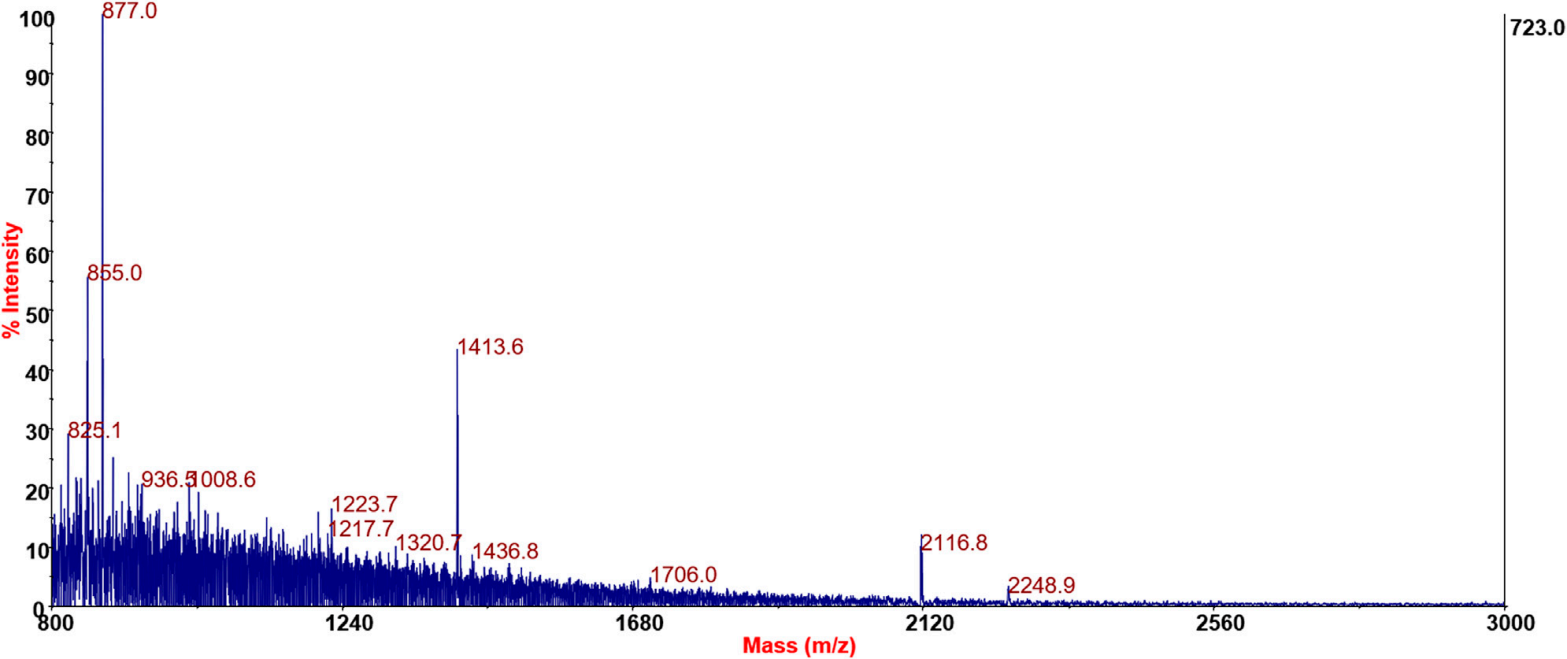
Positive reflector mode spectra of SPFs derived from LAC92.

Significant results for SPF samples’ MS/MS spectra containing low-mass amino acidic immonium ions and delta mass between fragments related to amino acid residues are shown in Figure 5.

### 3.4 Biological activity of SPFs upon long-term storage

Biological analysis was performed to evaluate the stability of SPFs over 6-month storage at +4°C and −30°C. Unfortunately, when stored at +4°C, the products lose all biological activities after only 30 days. On the other hand, we found that SPFs stored at −30°C showed biological activity comparable to that of the fresh product at all times tested (Supplementary Figure S1). These data indicate that long-term storage at −30°C did not impair the SPFs that remained remarkably constant during the experiments.

### 3.5 Amino acid structure of peptide 92

Since it was unable to fully identify the peptides linked to the peptidoglycan by chromatography, as previously suggested by Fichera et al. (2016), we proceeded with another type of investigation. We have searched for the gene *locus* related to the synthesis of peptidoglycans (so called *mur* genes). The list of *mur* genes (CDS region) and their products involved in PG synthesis is described in Supplementary Table S1. From this analysis, the sequence of the pentapeptide bound to muramic acid was presumed. The pentapeptide, defined as peptide 92, linked to the peptidoglycan fragment has the following structure:

l-Ala-d-Glu-2,6DAP-d-Ala-d-Ala

### 3.6 Docking calculations

**FIGURE 6:**
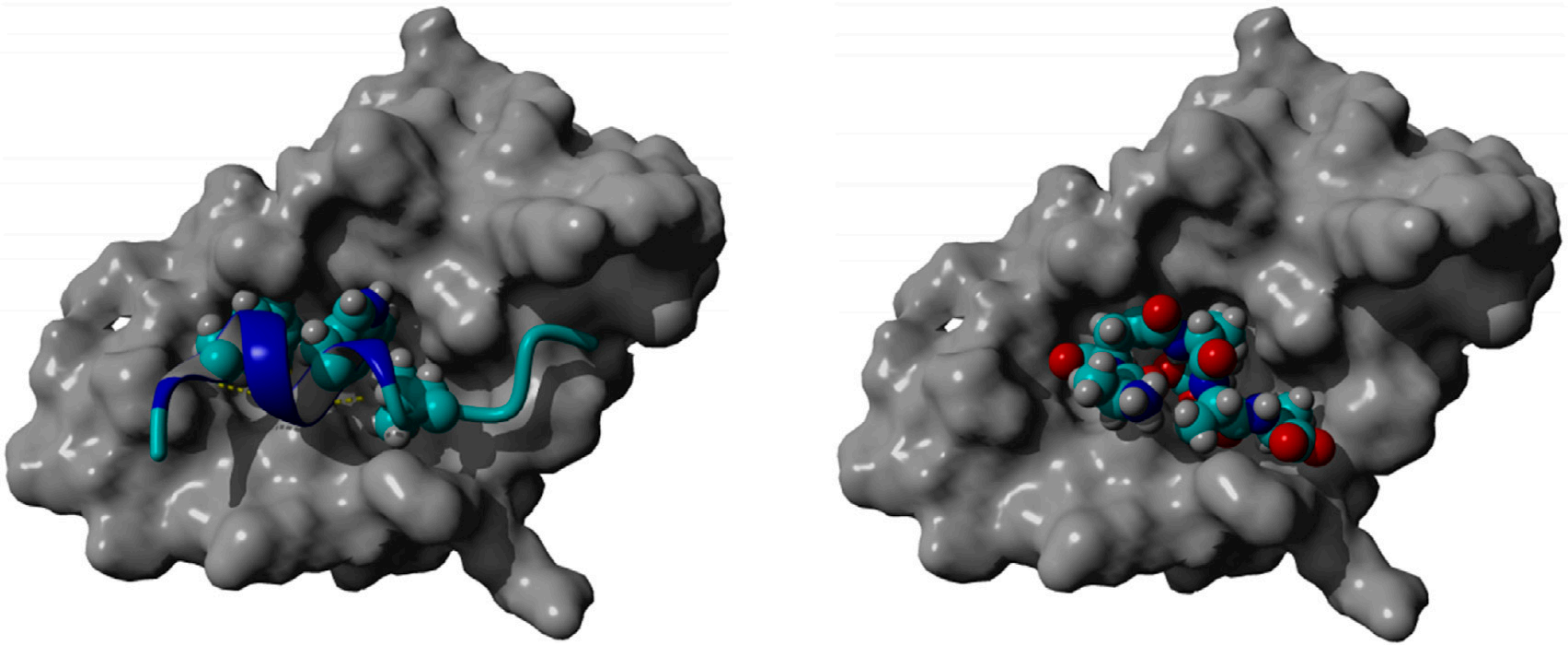
Crystallographic pose of p53 within the MDM2 groove (left); best-docked pose of peptide 92 within the MDM2 groove (right).

Molecular docking of peptide 92 revealed an interaction with the MDM2 protein, the negative regulator of p53 (Figure 6, left). By interacting with this protein, peptide 92 could aid the p53-driven induction of apoptosis. Indeed, docking studies performed with YASARA software show that peptide 92 allocates in the same groove as MDM2, which binds to p53 (Figure 6, right). Furthermore, the calculated *K*_i_ for p53 and peptide 92 of 185.5 µM and 62.7 µM, respectively, suggests that peptide 92 can displace p53 from the MDM2-binding site.

## 4 Discussion

Human microbiota has emerged as a key modulator of many disease treatment responses (Al-Qadami et al., 2022). In particular, it is already known that bacteria with probiotic properties, particularly *Lactobacillus*, can induce antitumor action by improving the apoptosis of tumor cells and protecting them from oxidative stress (Badgeley et al., 2021). In this regard, the gut microbiota has been involved in the efficacy of anti-PD1 and anti-PDL1 immunotherapies (Frankel et al., 2017; Matson et al., 2018; Peters et al., 2019), further confirming the crucial role of microbiota in cancer. Moreover, the antitumoral activity exerted by specific microbial species has been noticed in preclinical mouse models, suggesting that the specific presence of differential microorganisms due to co-housing or a fecal transplant was responsible for improvement in immune-checkpoint blockade agents (Sivan et al., 2015; Vetizou et al., 2015). Notably, the anticancer properties seem to be specifically related to peptidoglycan remodeling, which is involved in the dissemination of fragments into circulation, affecting immune response (Clarke et al., 2010) as well as the presence of biomarkers like NlpC/p60 hydrolases or NOD2-muropeptides. Based on this evidence, some papers suggest the importance of peptidoglycan remodeling enzymes in reprogramming probiotic bacteria to enhance the host’s immune response mechanisms (Griffin et al., 2021).

For a strain to be exploited in the therapeutic field, it is essential to define it as “probiotic” in terms of efficacy and safety. There are several criteria for recognizing bacteria as probiotics, such as their ability to survive in the GI-tract because of acidic conditions and bile salts, then being able to adhere to the intestinal cells, and, especially, have amensalistic properties (Fuochi et al., 2015; Stivala et al., 2021).

In this study, we aimed to investigate the antitumor effects of *Limosilactobacillus fermentum* strain against human colorectal cancer. In particular, the study targeted the effects of the peptidoglycan fragments released by the strain LAC92, for which the probiotic characteristics had already been proven (Fuochi et al., 2017). For this purpose, we screened the cytotoxicity and antiproliferative activity of LAC92 SPFs against human colorectal cancer HCT116 cells. Moreover, we analyzed the molecular structure of the peptidoglycan fragments to clarify the role of the peptide chains involved in the antitumor activity.

First of all, our observations confirmed amensalistic activity. Indeed, SPFs showed antibacterial activity against all pathogens tested. The best results were obtained against *S. lutea*, followed by *A. baumannii*, *K. pneumoniae*, and *E. coli*. Moreover, the iron-scavenging siderophore pyoverdine, a major virulence regulatory mechanism of *P. aeruginosa*, has also been inhibited. Nevertheless, the better activity shown by crude CFS than SPFs suggested the presence of other active molecules, which were probably lost during the peptidoglycan isolation process. Indeed, many LABs use narrow-spectrum toxins, such as bacteriocins, as a signature of ecological dominance (Fuochi et al., 2021a; Palmer and Foster, 2022). Moreover, *Lactobacillus* spp. synthesizes exopolysaccharides, which play an important role in preventing human diseases, thanks to their anticancer, immunomodulatory, and antimicrobial properties (Oleksy and Klewicka, 2018). Therefore, it is natural to hypothesize that molecules of different natures act synergistically by increasing the antibacterial effects demonstrated by CFS.

Based on the microbiological outcomes, we further investigated the role of SPFs in an *in vitro* model of a human colorectal cancer HCT116 cell line. In this regard, recent scientific literature widely focuses on the anticancer properties exerted by gut microbiota against colorectal cancer (Chen and Li, 2020; Sheikh et al., 2021). In particular, most of the clinical research between 2015 and 2017 shows the crucial role of specific gut microbiomes in postsurgical complications of colorectal cancer (Aisu et al., 2015; Consoli et al., 2016; Theodoropoulos et al., 2016; Flesch et al., 2017; Hibberd et al., 2017) acting on immune system functionality and inflammation.

The first preliminary analysis was based on selecting the proper SPF concentration of SPFs to perform all the biological investigations. In this regard, the data shown in Figure 1 showed the variable effects of SPFs on the cell index value in a concentration-dependent manner. The data obtained showed immediate cytotoxicity from 15%v/v upward. This evidence motivated us to select the first cytostatic but not cytotoxic concentration (13%v/v) to establish a proper model to evaluate further biological analyses. The subsequent investigations showed a substantial reduction in cell viability and the number of cells (Figure 2) after 24 (Figure 2A) and 48 h (Figure 2B).

Effectively, our data broadly coincided with scientific evidence showing the pro-apoptotic effect of peptidoglycan from *Lacticaseibacillus paracasei* in the cancer HT-29 cell line (Tian et al., 2015) through mechanisms involving endoplasmic reticulum damage. In addition, we obtained further encouraging results demonstrating the inhibitory properties of SPFs on cell migration, cell proliferation, and colony formation, as shown in Figure 3 and Figure 4.

Actually, PG derived from probiotics had encouraging antiproliferative activity on several cancer cell lines. Even though MS-MS could not assign a unique sequence to the peptide responsible for the excellent antiproliferative activity shown by SPFs deriving from LAC92, it was possible to confirm the amino acid structure involved in biological activity, thanks to the associated bacterial genomic sequence. These kinds of peptides are typically involved in the PG of *L. fermentum* (Barretto et al., 2016)*.* So far, *L. casei* is the strain that has mostly been proven to have immunomodulatory and antitumor effects (Lee et al., 2004; Jacouton et al., 2018; Kim et al., 2019; Dadfarma et al., 2021; Spyridopoulou et al., 2021), but also live *L. fermentum* strain exerted the ability to inhibit cancer cells but not normal colon cells and protected them from the damaging effect of a carcinogen (Kahouli et al., 2017).

Although one critical point should be noted, which is that *in vitro* methods do not accurately represent *in vivo* conditions, taken together, these data demonstrated a crucial role of SPFs of *L. fermentum* LAC92 in the viability and proper functionality of cancer cells. These suggested its potential anticancer activity. This possibility was further investigated *in silico* by studying the interaction of peptide 92 with the MDM2 protein, the negative regulator of p53. By interacting with this protein, peptide 92 could induce apoptosis driven by p53.

The crystal structure of the amino-terminal domain of MDM2 bound to a 15-residue transactivation domain peptide of p53 disclosed that MDM2 has a deep hydrophobic cleft on which the p53 peptide binds as an amphipathic *α*-helix (Kussie et al., 1996). The contact between the two proteins is based on a triad of p53 amino acids, Phe19, Trp23, and Leu26, which fit perfectly with the groove of MDM2 (Figure 6, left).

The hypothesis is that MDM2 inactivates p53 by hiding its transactivation domain, disrupting its interaction with the general transcription machinery. Therefore, targeting the MDM2–p53 interaction by small molecules to reactivate p53 has emerged as a promising new cancer therapeutic strategy (Wiman, 2006; Vassilev, 2007).

Docking studies, performed using the YASARA software, of peptide 92 with only MDM2, employing the crystal structure in which it is bound to the transactivation domain of p53 (PDB: 1YCR), show that peptide 92 allocates in the same groove as MDM2, which binds to p53 (Figure 6, right). Moreover, the calculated *K*_i_ for p53 and peptide 92 of 185.5 μM and 62.7 μM, respectively, suggests that peptide 92 may displace p53 from the MDM2-binding site.

Furthermore, the biological evidences obtained, combined with the encouraging notions offered by scientific literature, lead us to speculate that LAC92 should represent an important resource in colorectal cancer treatment, in combination or not with the traditional therapies. The next steps to consider are the total purification of the bioactive molecule and its evaluation on *ex vivo* models, and subsequently, on *in vivo* cancer models to obtain results more congruous to its therapeutic potential.

## Data Availability

The original contributions presented in the study are included in the article/Supplementary Materials, further inquiries can be directed to the corresponding author.
