# Supplementary material for: Soluble peptidoglycan fragments produced by Limosilactobacillus fermentum with antiproliferative activity are suitable for potential therapeutic development: A preliminary report
Source: Front Mol Biosci. 2023 Feb 15;10:1082526. doi: 10.3389/fmolb.2023.1082526 (PMC9975264; doi:10.3389/fmolb.2023.1082526)
Supplement: Supplementary file 1 [file Table1.DOCX]

Supplementary Material

**Soluble peptidoglycan fragments produced by *Limosilactobacillus fermentum* with antiproliferative activity are suitable for potential therapeutic development - A preliminary report**

Virginia Fuochi^1,2*^, Mariarita Spampinato^1^, Alfio Distefano^1^, Angelo Palmigiano^3^, Domenico Garozzo^3^, Chiara Zagni^4^, Antonio Rescifina^4^, Giovanni Li Volti^1,2^ And Pio M. Furneri^1,2^

**Correspondence:** Virginia Fuochi: vfuochi@unict.it

# Biological Activity of SPFs upon Long-Term Storage

MIC values were determined by microdilution broth method, as already described, to evaluate the SPFs stability in terms of antimicrobial efficiency over the 6-month storage at +4°C and -30°C. Unfortunately, when stored at +4°C the products lose almost all antimicrobial activities after only 30 days. On the other hand, we found that SPFs stored at -30°C showed efficiency comparable to the fresh product at all times tested (Fig S1).


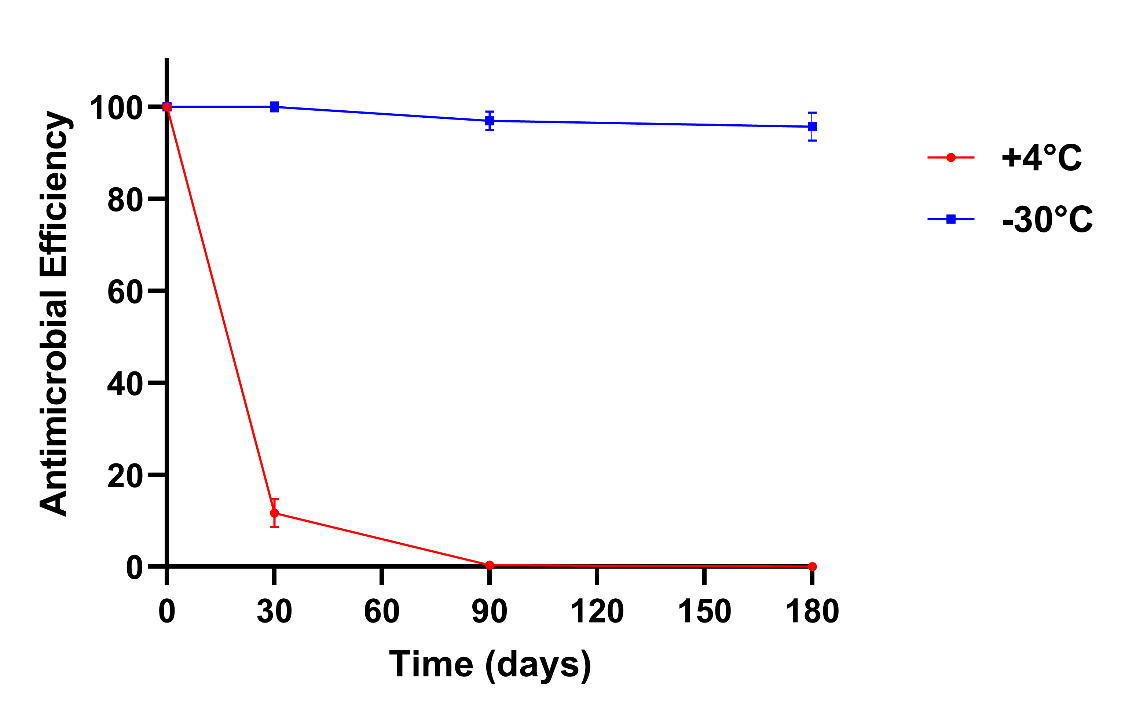


**Supplementary Figure 1.** Antimicrobial efficiency of SPFs tested against *E. coli*. SPFs were stored for a maximal time of 6-months at +4°C and -30°C. Antibacterial activity was tested at 0, 30, 90 and 180 days. Data were expressed as mean ± SD (n = 3). The differences between data at day 0 and 180 after storage at -30°C were statistically not significant (p > 0.05).

**Table S1. *Limosilactobacillus fermentum* strain LAC FRN-92 chromosome, complete genome NCBI Reference Sequence: NZ_CP021790.1: list of *mur* genes (CDS region) and their product involved in PG synthesis**

| ***mur* gene** | **CDS** | **complement** | **Product** |
| --- | --- | --- | --- |
| *mur*A | 731896..733164 | yes | UDP-N-acetylglucosamine 1-carboxyvinyltransferase |
| *mur*A | 272088..273311 | yes | UDP-N-acetylglucosamine 1-carboxyvinyltransferase |
| *mur*B | 1535875..1536771 | yes | UDP-N-acetylmuramate dehydrogenase |
| *mur*C | 354245..355558 | no | UDP-N-acetylmuramate--L-alanine ligase |
| *mur*D | 853701..855077 | no | UDP-N-acetylmuramoyl-L-alanine--D-glutamate ligase |
| *mur*D *alternative* | 337704..338984 | no | UDP-N-acetylmuramoyl-L-alanine-D-glutamate ligase) |
| *mur*E | 710904..712460 | yes | UDP-N-acetylmuramoyl-L-alanyl-D-glutamate-2, 6-diaminopimelate ligase [EC 6.3.2.13]* |
| *mur*E *alternative* | 712465..714000 | yes | UDP-N-acetylmuramyl-tripeptide synthetase |
| *mur*E  *putative* | 476830..478167 | yes | Mur ligase family protein 98% homology to *mur*E product [PDB entry: [1E8C](https://www.rcsb.org/structure/1E8C)] |
| *mur*F | 1124933..1126312 | yes | UDP-N-acetylmuramoyl-tripeptide-D-alanyl-D-alanine ligase [EC 6.3.2.10] |
| *mur*G | 855077..856183 | no | undecaprenyldiphospho-muramoylpentapeptide beta-N-acetylglucosaminyltransferase |
| *mur*I | 207085..207891 | yes | glutamate racemase |

*The presence of gene *dap*F - EC 5.1.1.7 (CDS region 1570026..1571033) is related to EC 6.3.2.13 according to “KEGG lysine synthesis pathways” accessed on January 24^th^, 2023 [https://www.genome.jp/pathway/map00300+K01928]
